# Supplementary material for: Redosing with Intralymphatic GAD-Alum in the Treatment of Type 1 Diabetes: The DIAGNODE-B Pilot Trial
Source: Int J Mol Sci. 2025 Jan 4;26(1):374. doi: 10.3390/ijms26010374 (PMC11720063; doi:10.3390/ijms26010374)
Supplement: Supplementary file 1 [file ijms-26-00374-s001.zip › ijms-3362791-supplementary.pdf]

International Journal of *Molecular Sciences*

MDPI

Supplementary Tables

Table S1. C-peptide $AUC_{0\text{-}120\ min}$ during MMTT per visit.

Visit	Statistics	Actual (N=6)	Change from baseline (N=6)
Month 0	Mean (CVGeo)	0.083 (1.586)	-
(Baseline)	Median (min:max)	0.093 (0.019:0.308)	-
Month 6	Mean (CVGeo)	0.053 (2.159)	0.635 (0.356)
	Median (min:max)	0.065 (0.010:0.214)	0.681 (0.333:0.878)
Month 12	Mean (CVGeo)	0.050 (2.746)	0.607 (0.405)
	Median (min:max)	0.056 (0.010:0.266)	0.636 (0.333:0.895)

Actual values are expressed as nmol/L/min on logarithmic scale. Logarithmic values were back transformed to original (geometric) scale and changes from baseline are expressed as ratios (of geometric means). Abbreviations: - = not applicable; AUC = area under the curve (normalized to 120 min.); CVGeo = geometric coefficient of variation; max = maximum; MMTT = mixed meal tolerance test; min = minimum; N = number of patients.

Table S2. HbA1c levels per visit (mmol/mol).

Visit	Statistics	Actual (N=6)	Change from baseline (N=6)
Month 0	Mean (SD)	53.0 (10.3)	-
(Baseline)	Median (min:max)	48.5 (43:7)	-
Month 6	Mean (SD)	53.0 (10.3)	0.0 (3.2)
	Median (min:max)	48.5 (43:67)	0.0 (-4:4)
Month 12	Mean (SD)	55.3 (10.4)	2.3 (4.1)
	Median (min:max)	54.0 (43:7)	3.5 (-4:7)

Abbreviations: - = not applicable; max = maximum; min = minimum; N = number of patients; SD = standard deviation.

Table S3. Daily exogenous insulin consumption reported by patients, per visit (IU/kg/day).

Visit	Statistics	Actual (N=6)	Change from baseline (N=6)
Month 0	Mean (SD)	0.701 (0.18)	-
(Baseline)	Median (min:max)	0.658 (0.47:1.02)	-
Month 6	Mean (SD)	0.529 (0.27)	-0.172 (0.18)
	Median (min:max)	0.464 (0.31:1.06)	-0.144 (-0.42:0.04)
Month 12	Mean (SD)	0.553 (0.13)	-0.147 (0.17)
	Median (min:max)	0.546 (0.36:0.76)	-0.216 (-0.28:0.14)

Abbreviations: - = not applicable; max = maximum; min = minimum; N = number of patients; SD = standard deviation.

Table S4. Insulin-dose-adjusted HbA1c (IDAAc) levels per visit.

Visit	Statistics	Actual (N=6)	Change from baseline (N=6)
Month 0	Mean (SD)	9.8 (1.1)	-
(Baseline)	Median (min:max)	9.4 (8.8:11.8)	-
Month 6	Mean (SD)	9.1 (1.8)	-0.7 (0.8)
	Median (min:max)	8.5 (7.3:12.3)	-0.5 (-1.7:0.5)
Month 12	Mean (SD)	9.4 (1.4)	-0.4 (0.8)
	Median (min:max)	9.3 (7.5:11.3)	-0.4 (-1.5:0.8)

Abbreviations: - = not applicable; max = maximum; min = minimum; N = number of patients; SD = standard deviation.

Table S5. Time in glycaemic target range 3.9 to 10 mmol/L (70 to 180 mg/dL).

Visit	Statistics	Actual (N=6)	Change from baseline (N=6)
**Sum of time (hours) in glycaemic target range**			
Month 0 (Baseline)	Mean (SD)	182.0 (52.0)	-
	Median (min:max)	186.4 (125.3:260.8)	-
Month 6	Mean (SD)	184.1 (71.6)	2.2 (45.5)
	Median (min:max)	205.9 (54.3:248.5)	22.3 (-71.0:45.5)
Month 12	Mean (SD)	194.9 (49.7)	12.9 (66.4)
	Median (min:max)	194.6 (142.0:259.3)	29.4 (-118.8:58.8)
**Percentage of time spent in glycaemic range**			
Month 0 (Baseline)	Mean (SD)	60.7 (15.2)	-
	Median (min:max)	62.4 (37.4:78.0)	-
Month 6	Mean (SD)	58.3 (21.5)	-2.4 (13.0)
	Median (min:max)	67.0 (16.2:74.3)	-1.1 (-21.2:13.6)
Month 12	Mean (SD)	68.6 (10.6)	7.9 (10.1)
	Median (min:max)	71.3 (48.1:77.5)	7.4 (-2.7:21.4)

Abbreviations: - = not applicable; max = maximum; min = minimum; N = number of patients; SD = standard deviation.

Table S6. Time in hyperglycaemic range >10 mmol/L (>180 mg/dL).

Visit	Statistics	Actual (N=6)	Change from baseline (N=6)
**Sum of time (hours) in the hyperglycaemic range**			
Month 0 (Baseline)	Mean (SD)	102.1 (61.9)	-
	Median (min:max)	103.1 (28.0:209.3)	-
Month 6	Mean (SD)	110.5 (89.1)	8.4 (37.7)
	Median (min:max)	90.9 (14.3:280.3)	-3.4 (-29.8:71.0)
Month 12	Mean (SD)	75.3 (52.5)	-26.8 (25.5)
	Median (min:max)	55.1 (37.5:173.5)	-26.9 (-60.0:13.8)
**Percentage of time spent in hyperglycaemic range**			
Month 0 (Baseline)	Mean (SD)	32.9 (17.8)	-
	Median (min:max)	33.7 (12.3:62.6)	-
Month 6	Mean (SD)	34.1 (26.5)	1.3 (11.9)
	Median (min:max)	27.2 (5.1:83.8)	-3.7 (-8.9:21.2)
Month 12	Mean (SD)	25.2 (13.8)	-7.7 (8.9)
	Median (min:max)	20.7 (13.1:51.9)	-8.8 (-20.2:3.8)

Abbreviations: - = not applicable; max = maximum; min = minimum; N = number of patients; SD = standard deviation.

Table S7. Serum GAD_{65} antibody titers (U/ml) per patient.

Subject for the Study	Sex	GADA 0 month	GADA 1 month	GADA 3 month	GADA 6 month	GADA 12 month
A1	Male	106600	226100	101300	70300	131300
A2	Male	166400	136600	225500	379800	265700
B1	Female	5800	6810	147800	42700	49720
B2	Male	22470	20640	37020	55400	35280
B3	Male	12090	10300	69700	38080	32150
B4	Female	228400	156900	294200	149500	166300

Table S8. Serum GADA titers (U/mL) per visit.

Visit.	Statistics	Actual (N=6)	Change from baseline (N=6)
Month 0 (Baseline)	Mean (SD)	92892 (92956)	-
	Median (min:max)	78620 (6810:226100)	-
Month 3	Mean (SD)	145920 (97962)	53028 (99129)
	Median (min:max)	124550 (37020:294200)	74150 (-124800:140990)
Month 6	Mean (SD)	122630 (132404)	29738 (127622)
	Median (min:max)	62850 (38080:379800)	31270 (-155800:243200)
Month 12	Mean (SD)	113408 (92815)	20517 (71763)
	Median (min:max)	90510 (32150:265700)	18245 (-94800:129100)

Abbreviations: - = not applicable; max = maximum; min = minimum; N = number of patients; SD = standard deviation.

Supplementary Figure

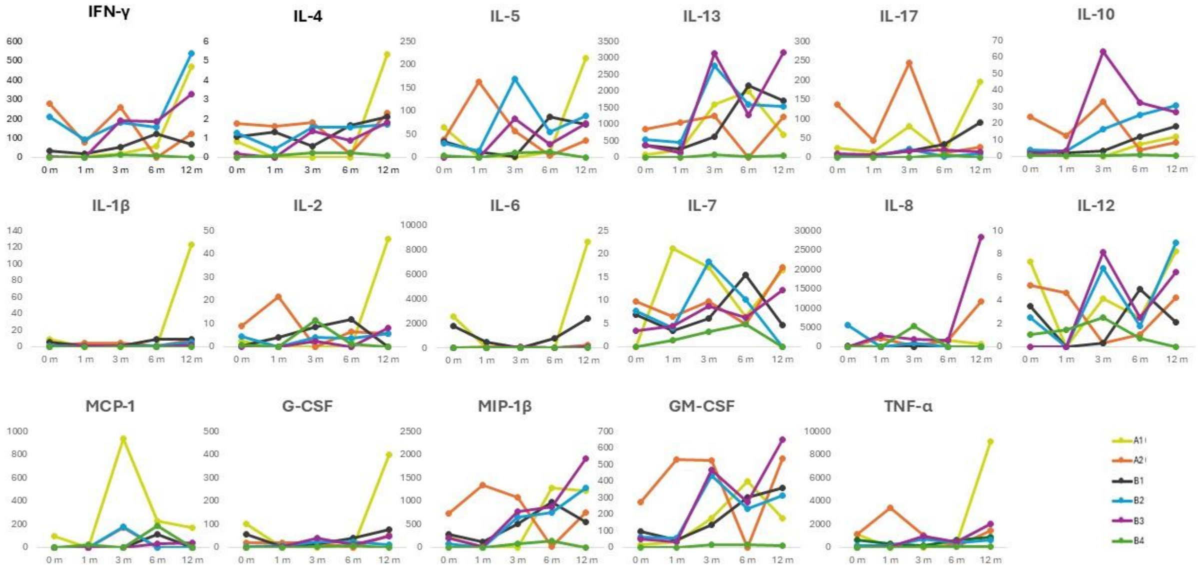

Figure S1. The GAD_{65} induced cytokine secretion upon in vitro PMBC stimulation in patients (A1, A2, B1, B2, B3, B4) who received a late booster injection of GAD-alum. The cytokines IL-1b, IL-2, IL-4, IL-5, IL-6, IL-7, IL-8, IL-10, IL-12, IL-13, IL-17, tumor necrosis factor (TNF-α), interferon (IFN-γ), and MCP-1, G-CSF, MIP-1β, GM-CSF chemokines were detected by Luminex in supernatants collected after 7 days culture. GAD_{65}-induced cytokine secretion is given as pg/mL after subtraction of spontaneous secretion. The results are based on samples collected at start of DIAGNODE-B (0 months), before the GAD-alum redosing (1 months (0 m)) and after 3, 6 and 12 months (m).
